# Web-based LinRegPCR: application for the visualization and analysis of (RT)-qPCR amplification and melting data

**DOI:** 10.1186/s12859-021-04306-1

**Published:** 2021-08-24

**Authors:** Andreas Untergasser, Jan M. Ruijter, Vladimir Benes, Maurice J. B. van den Hoff

**Affiliations:** 1 Center for Molecular Biology of Heidelberg University (ZMBH), 69120 Heidelberg, Germany; 2 European Molecular Biology Laboratory (EMBL), Genomics Core Facility, 69117 Heidelberg, Germany; 3 Department of Medical Biology, Amsterdam University Medical Centres, Academic Medical Center, Meibergdreef 15, 1105 AZ Amsterdam, The Netherlands

**Keywords:** LinRegPCR, RDML, qPCR, PCR, Amplification curve, Melting curve

## Abstract

**Background:**

The analyses of amplification and melting curves have been shown to provide valuable information on the quality of the individual reactions in quantitative PCR (qPCR) experiments and to result in more reliable and reproducible quantitative results.

**Implementation:**

The main steps in the amplification curve analysis are (1) a unique baseline subtraction, not using the ground phase cycles, (2) PCR efficiency determination from the exponential phase of the individual reactions, (3) setting a common quantification threshold and (4) calculation of the efficiency-corrected target quantity with the common threshold, efficiency per assay and C_q_ per reaction. The melting curve analysis encompasses smoothing of the observed fluorescence data, normalization to remove product-independent fluorescence loss, peak calling and assessment of the correct peak by comparing its melting temperature with the known melting temperature of the intended amplification product.

**Results:**

The LinRegPCR web application provides visualization and analysis of a single qPCR run. The user interface displays the analysis results on the amplification curve analysis and melting curve analysis in tables and graphs in which deviant reactions are highlighted. The annotated results in the tables can be exported for calculation of gene-expression ratios, fold-change between experimental conditions and further statistical analysis. Web-based LinRegPCR addresses two types of users, wet-lab scientists analyzing the amplification and melting curves of their own qPCR experiments and bioinformaticians creating pipelines for analysis of series of qPCR experiments by splitting its functionality into a stand-alone back-end RDML (Real-time PCR Data Markup Language) Python library and several companion applications for data visualization, analysis and interactive access. The use of the RDML data standard enables machine independent storage and exchange of qPCR data and the RDML-Tools assist with the import of qPCR data from the files exported by the qPCR instrument.

**Conclusions:**

The combined implementation of these analyses in the newly developed web-based LinRegPCR (https://www.gear-genomics.com/rdml-tools/) is platform independent and much faster than the original Windows-based versions of the LinRegPCR program. Moreover, web-based LinRegPCR includes a novel statistical outlier detection and the combination of amplification and melting curve analyses allows direct validation of the amplification product and reporting of reactions that amplify artefacts.

**Supplementary Information:**

The online version contains supplementary material available at 10.1186/s12859-021-04306-1.

## Background

The analysis of fluorescence data generated by real-time monitoring of PCR reactions [1] is most often done with the software available in the qPCR machine. In general, the qPCR machine subtracts a baseline fluorescence, sets a quantification threshold and reports the C_q_ value, which is the number of cycles required to reach that threshold [2]. Many users report only this C_q_ value as outcome of their qPCR study [3]. More advanced users derive the PCR efficiency from a standard curve [4] and calculate efficiency-corrected outcomes for their qPCR experiments [5]. Already in the beginning of this millennium the PCR efficiency values derived from standard curves were found to be different between machines and, most troubling, difficult to reproduce between PCR runs. Therefore, several methods to analyse amplification curves were proposed [6–13]. These methods not only report a C_q_ value but also derive an efficiency value and some quality measures from each amplification reaction. A comparison of these methods showed that the amplification curve analysis performed by LinRegPCR achieved qPCR results with the lowest variation and highest reproducibility [14].

Monitoring of the PCR reaction with DNA-binding fluorochromes is commonly used in experimental biological applications in which many and often changing targets are measured. However, with these dyes the amplification of artefacts leads to amplification curves that are indistinguishable from those of the correct target [15]. Melting curve analysis can then be used to check whether only the correct product or more products are amplified [16]. Moreover, it was shown that the melting curve analysis can be used to determine the contribution of different products to the amplification curve and thus to correct the reported qPCR result when artefacts are amplified [17].

### Amplification curve analysis

**Fig. 1:**
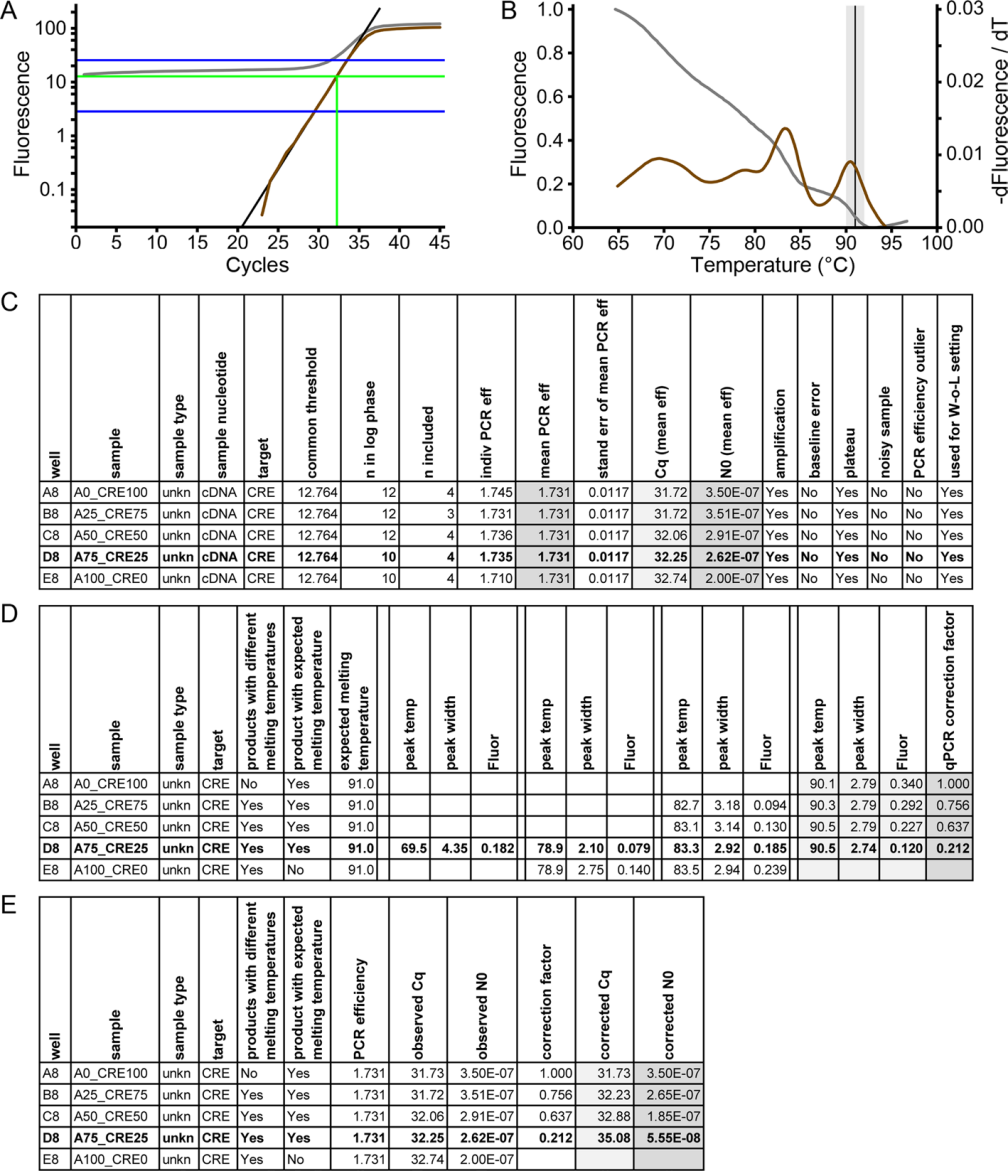
Summary of the output of web-based LinRegPCR. **A** Amplification data of well D8 with sample A75_CRE25. The grey curve shows the raw data, the brown curve the baseline-corrected data. The W-o-L is restricted by the blue lines and serves to select the data points from which the PCR efficiency of the reaction is determined. The quantification threshold and the called C_q_ are shown in green. The black line is the associated idealized curve based on the mean PCR efficiency of the assay. **B** Melting data of well D8 with sample A75_CRE25. The grey curve shows the normalized melting data. The brown curve is the negative first derivative data and shows the melting peaks. The grey area highlights the expected temperature range with the expected temperature provided by the user (T_m_) as black line. The peak of the amplified product (observed T_m_) in this reaction falls within the grey range, indicating that the amplified product represents the intended product. **C** The result table of the amplification curve analysis adapted for publication. **D** The result table of melting curve analysis. In contrast to the web version the first columns, showing the peak of the intended target, were removed and the identical columns were highlighted by a grey background. **E** Calculation of the corrected N_0_ and C_q_ values using the N_0_ and C_q_ resulting from the amplification curve analysis and the correction factor found in the melting curve analysis

The amplification curve comprises of four distinct phases; 1) the ground phase in which amplification-dependent fluorescence is below the measurement noise, 2) the exponential phase with monotonically increasing fluorescence values and constant PCR efficiency, 3) the transition phase, in which the PCR efficiency decreases because of limiting reaction components and 4) the plateau phase where amplification stops and fluorescence remains constant [18, 19]. In a logarithmic plot of the amplification curve, the exponential phase of the reaction is a straight line, the slope of which is determined by the PCR efficiency (Fig. 1A, black line). Analysis of individual amplification curves requires the following steps: (1) baseline subtraction, (2) identification of the exponential phase, (3) determining the PCR efficiency, (4) calling the C_q_ value, and (5) calculating the target quantity.

Baseline fluorescence is defined as the observed fluorescence that is independent of amplification. This baseline fluorescence is mainly the result of incomplete quenching of the fluorophore in hydrolysis probe assays and of unbound dye in DNA-binding dye assays. Nonspecific primer annealing and binding of probes or fluorochrome to genomic DNA contamination can also result in measurable baseline fluorescence. In optimized DNA dye-based assays, baseline fluorescence is below 1% of the fluorescence at the end of the PCR run; in probe-based assays the baseline may still be as high as 10% of the observed fluorescence. Almost all qPCR machines calculate a trend line through the fluorescence values of the so-called ground phase cycles and extrapolate this baseline trend to the last cycle. This system baseline is thus based on the lowest, most noisy, fluorescence values. Moreover, the option to manually set, or change, the ground phase opens the door for user bias [20]. Over- or underestimation of the baseline fluorescence strongly affects the slope of the exponential phase of the baseline-corrected amplification curve and thus the PCR efficiency determined from this slope (see below). In both the windows and web-based LinRegPCR an automated baseline estimation is implemented that is user independent and does not use the ground phase measurements. This baseline determination uses an iterative approach that determines a baseline value that leaves the most data points on a straight line in a log(fluorescence) versus cycle number plot [13]. Comparison of the raw fluorescence data (Fig. 1A, grey curve) with the baseline-corrected data of the same reaction (Fig. 1A, brown curve) shows the reconstruction of the straight exponential phase. An additional file illustrates the observed and baseline-corrected fluorescence data on linear and logarithmic fluorescence scales [see Additional file 1].

To identify the exponential phase in the baseline-corrected amplification curve, the subset of cycles has to be identified that show a continuous increase of fluorescence values. The start of this exponential phase is defined as the first cycle after which the increase in the fluorescence per cycle is continuous and the exponential phase ends where this increase starts to decrease; the latter is marked by the so-called second derivative maximum (SDM) [4]. After the SDM the reaction enters the transition phase.

The PCR efficiency value for each reaction is determined from at least three consecutive cycles in the exponential phase. Because there is still some effect from residual baseline noise, the PCR efficiency is calculated by averaging all PCR efficiencies determined from the individual reactions of the same target, which results in the least variable mean PCR efficiency per assay [14]. This approach to determine the PCR efficiency is superior to the classically used standard curve, as recently reviewed [3].

The C_q_ value, or quantification cycle [2], is the fractional number of cycles that is required to reach the quantification threshold (N_q_). To provide a valid C_q_ value, the quantification threshold should be placed in the exponential phase whereas direct comparison of C_q_ values requires that the threshold is the same for all reactions and assays in the run [20]. Many qPCR systems set the quantification threshold at 10-times the standard deviation of the noise in the ground phase values. Because of the highly variable noise in the first cycles of the PCR, these thresholds are different between runs and thus not standardized. To standardize the threshold setting, web-based LinRegPCR sets a common threshold for all assays in the exponential phase of all reactions (see  implementation). The C_q_ value per reaction is mathematically determined as the intersection of the threshold with the straight line through the exponential phase of each reaction; the cycle axis value corresponding with this intersection is reported as C_q_. Although a common threshold allows direct comparison of C_q_ values between reactions, C_q_ also depends heavily on the PCR efficiency of the assay and, therefore, reporting of C_q_ values is not recommended [2].

To by-pass biases resulting from interpretation of reported C_q_ values [3], LinRegPCR reports the efficiency-corrected target quantity (N_0_) per reaction calculated with the quantification threshold, the PCR efficiency of the assay and the C_q_ value of the reaction [13] (Fig. 1C). With easy mathematics, these target quantities can then be used to calculate gene-expression ratios (ratio = N_0,target_/N_0,reference_) and fold-difference in gene expression between experimental conditions (fold = ratio_experiment_/ratio_control_) [3] [21].

### Melting curve analysis

Most qPCR systems can perform a melting protocol after the amplification protocol. The melting protocol involves gradual heating of the reaction volume while continuously monitoring the fluorescence. Heating will cause the double-stranded DNA to denature when its melting temperature (T_m_) is reached. When the DNA becomes single stranded, the bound fluorochrome is released and the observed fluorescence drops (Fig. 1B, grey curve). The T_m_ of a DNA fragment is determined by not only its length and GC content but also by its sequence context, PCR mix composition and ramp speed of the temperature gradient of the melting protocol [16]. To facilitate interpretation of the melting curve, the negative first derivative of the fluorescence data is calculated and plotted against the temperature range [22] (Fig. 1B, brown curve). In this plot, each amplification product will be visible as a different peak at the melting temperature of the associated product (T_m_) [17]. Comparison of the observed T_m_ to the T_m_ of the positive control, or the previously determined T_m_ of the correct product, enables discrimination between the correct amplification product and artifacts. When a saturating DNA-binding dye, e.g. LCGreen, is used, the fluorescence associated with the peak of the correct product can be used to remove artifact bias [17].

A complete melting curve analysis involves data processing, peak finding, identification of the melting peaks, measurement of the fluorescence associated with each peak and calculation of the contribution of the peak of the correct product to the total fluorescence. However, most, if not all, companies do not disclose how they perform analysis of melting curve data. Without going into an extensive review, we illustrate three examples. The Biorad CFX system has the user set a threshold to determine the minimum height of the melting peaks to be reported. The Roche Lightcycler system uses an undisclosed method to restrict the number of peaks reported to the number requested by the user. The ABI Prism system has the user set a temperature window in which the melting peak of the correct amplification product is expected and then evaluates the validity of the reaction by determining whether the peak area ratio of peaks inside and outside this window is above a user defined value. In all cases the systems report the T_m_ and other peak characteristics, like peak height, width and area. None of the systems attaches consequences of this analysis with respect to the reported quantitative data resulting from the amplification curve analysis. In the web-based LinRegPCR an unbiased transparent melting peak analysis procedure is included that is free of user involvement with respect to peak finding and identification [17]. The user can opt to exclude the respective reaction from further analysis and to remove the artefact bias from the reported quantitative results (N_0_) of the target.

### Web based implementation

LinRegPCR has been available for qPCR data analysis since 2003 [6], with a major update in 2009 [13]. A drawback of this implementation has always been that it could only be used in a Windows environment and for each different or updated qPCR machine export an import format needed to be created. Moreover, its slow performance, especially with the increase in plate size in the qPCR machines, became an annoyance for some users. This slowness is due to the graphic user interface that sits at the core of the LinRegPCR program. Instead of redesigning a Windows version from scratch, we decided to implement a web-based version of this qPCR amplification curve analysis program, thus achieving platform independence and speedier performance by optimizing the program code and separating the graphics interface from the data processing. As input LinRegPCR uses and takes advantage of the RDML file format which provides machine independent storage and exchange of qPCR data. Moreover, the melting curve analysis has been implemented in this novel application and integrated into the reported quantitative results.

## Implementation

### Implementation of amplification curve analysis

The first, and critical, step in the analysis is the accurate estimation of the baseline fluorescence per reaction. Both LinRegPCR versions determine this baseline in an iterative approach. Initially, the baseline is set to a level that is too high. Then the data points in the exponential phase are split into two parts and the slopes of the straight lines through the subsets are compared. The baseline estimate is step-wise lowered until the slope of the upper halve becomes steeper than the slope of the lower halve, indicating that the baseline estimate has become too low. The baseline estimate is then increased by one step and the step is halved. The procedure is iterated until the slopes differ less than 0.0001; at a PCR efficiency of 1.8, this criterion translates into an efficiency difference of 0.0004 [13]. This baseline correction thus reconstructs the exponential phase of the amplification curve. Failure to find a baseline value that gives a straight line through the exponential phase is reported as a baseline error.

The second step of the analysis is to determine the efficiency value from the slope of the line fitted to a subset of data points in the linear exponential phase of each individual reaction [6]. An initial high window of linearity (W-o-L) is set and for the valid reactions the individual PCR efficiencies are determined from the data points within this W-o-L. Per assay, the coefficient of variation (CV = standard deviation / mean) of the individual efficiencies is calculated and the W–o-L is shifted downward until the lowest CV is reached (Fig. 1A, cycles between the blue lines). The PCR efficiency for the assay is calculated as the mean of these individual PCR efficiencies. This procedure is performed for every assay present in the qPCR run. Reactions without amplification are always excluded before setting of the W–o-L. By default, reactions that do not reach a plateau are also excluded; the user can choose to include them.

The LinRegPCR web-application implements two strategies to determine, and optionally exclude, reactions with an individual PCR efficiency that deviates strongly from the mean efficiency per assay. In the Windows version of LinRegPCR the user has the option to use all PCR efficiencies or to exclude PCR efficiencies outside a user-defined range (default setting ± 0.05) around the median efficiency per assay. However, this simple approach tends to exclude too many reactions in wide normal distributions. To avoid this unwanted behaviour, a novel statistical outlier detection, based on the distribution skewness and the Grubbs’s test, is implemented in this new version of LinRegPCR. Because this optional outlier exclusion changes the distribution of the efficiencies of the included reactions, the W-o-L setting and mean efficiency calculation are iterated until no new outliers occur. Reactions considered to be efficiency outliers are reported in the result file (Fig. 1C).

The final steps in the amplification curve analysis are to call the C_q_ value and to calculate the efficiency-corrected target quantity (N_0_) per reaction. These steps require a quantification threshold to be set (Fig. 1A, horizontal green line); the C_q_ value is the cycle axis position of the intersection of the threshold with the amplification curve (Fig. 1A, vertical green line). Although not recommended [2], qPCR papers often only report C_q_ values. To compare such C_q_ values a common quantification threshold has to be set for all assays. For visualisation purposes, web-based LinRegPCR sets this common threshold in the exponential phase of all reactions in the run. To cancel out the random variation in individual PCR efficiencies, web-based LinRegPCR, determines for each reaction the centre of the exponential phase from the baseline-corrected fluorescence values and, using the mean PCR efficiency of the assay, constructs an ideal amplification curve which is then used to call the C_q_ value for the reaction. Additional files show the amplification curve analysis interface [see Additional file 2] and analysis results [see Additional file 3]. Note that the Windows version of LinRegPCR sets a quantification threshold per assay [13] and that, therefore, the reported C_q_ values cannot be directly compared or used in ΔC_q_ reports [3, 21].

The use of a common threshold for all assays does not remove the bias inherent to the fact that C_q_ values are efficiency-dependent [5]. Therefore, we strongly recommend the reporting of target quantities (N_0_ values) that are efficiency-corrected and can thus be freely compared between assays and runs [3]. The LinRegPCR program calculates these N_0_ values as ${N}_{0}={N}_{q}/{E}_{tar}^{{C}_{q}}$ with the common quantification threshold (N_q_), the PCR efficiency per assay (E_tar_) and the C_q_ value per reaction [13]. After amplification curve analysis, the program reports the target quantity per reaction as well as a number of quality measures based on the amplification curve. An additional file shows that, despite the different quantification thresholds and C_q_ values, the reported target quantities per reaction are the same for the two versions of LinRegPCR [see Additional file 1].

### Implementation of melting curve analysis

The measurement of the decreasing fluorescence when the temperature of the reaction is gradually increased, results in melting curve data (Fig. 1B, grey curve). The negative first derivative of these data reveals a peak, or peaks, of which each maximum represents the melting temperature (T_m_) of the associated DNA fragment(s) (Fig. 1B, brown curve). The second derivative of these data provides information on the width of each of these melting peak(s). To remove the measurement noise that complicates the identification of peaks in the first and second derivative, a good smoothing algorithm, that does not displace the position of the peaks, is essential [17]. To this end, a simplified version of Friedman’s supersmoother was used [23].

After smoothing, the melting data need to be normalized to remove the temperature-dependent but dissociation-independent fluorescence decrease [22]. In web-based LinRegPCR, the user can choose between bilinear, exponential or combined normalisation approaches. The bilinear normalization approach fits straight trend lines to subsets of data points in the lower and higher temperature ranges; the normalized melting curve is then calculated from the height of the observed melting curve above the lower trend line as a proportion of the difference between the upper and lower trend lines. The exponential normalization approach fits an exponential function based on the slopes of the melting curve at given temperatures at the start and the end of the temperature range. The combined normalization approach runs a bilinear normalization after the exponential normalization [17].

**Fig. 2:**
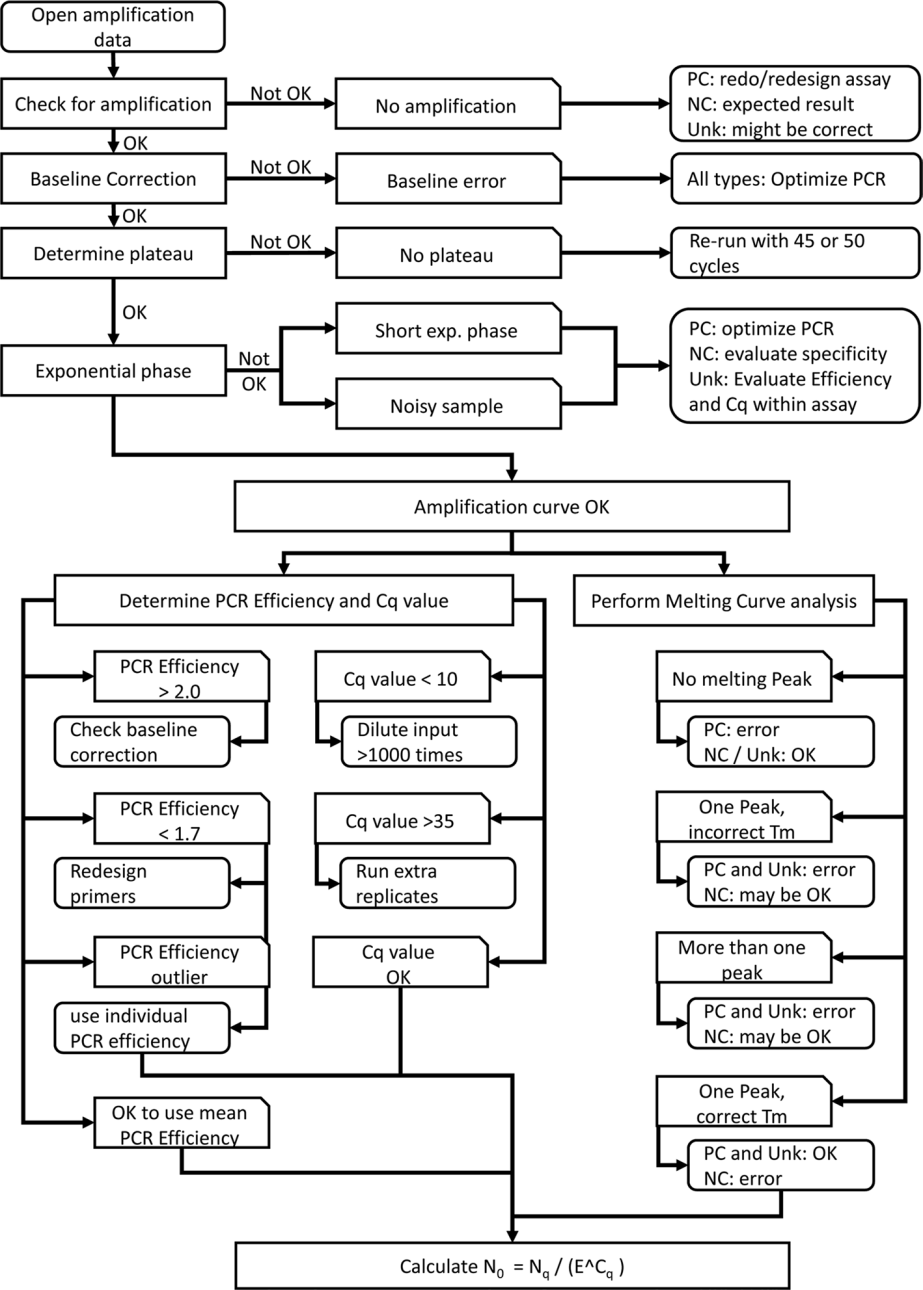
Illustration of the flow of the amplification and melting curve analysis. At different steps in data processing and analyses (rectangular boxes) the program can detect deviating reactions and provide warnings or errors (tabbed boxes). The flow chart gives a suggestion on how the user can deal with those warnings or on how to improve the assay to avoid these errors in the future (rounded boxes). Note that these recommendations are not exhaustive; basic knowledge on qPCR assay design and data analysis, as well as papers on these subjects, should guide the user in the appropriate direction. PC: positive control; NC: negative control; Unk: unknown sample;

Subsequently, the first and second derivative of the normalized data are calculated and used to identify melting peaks. The peak position, or melting temperature (T_m_), is then compared to the given melting temperature of the correct product, to assess whether the melting peak represents the correct product (Fig. 1B; vertical black line) or, if not, is associated with an artefact. The melting curve analysis algorithm uses the melting temperature of the intended target given in the RDML file to perform this assessment and gives appropriate error and warning messages when melting products with a deviating T_m_ are identified (Fig. 2). The program gives a warning when no product with the expected melting temperature is found and reports a note if more than one product is detected (Fig. 2 describes the handling of these errors and warnings). By default, peaks that have a melting temperature that is within 1.0 °C from the expected temperature of the target are considered to represent the correct product (Fig. 1B; grey area). This variation in T_m_ of the correct product was observed to occur in a study with 93 different validated targets with different T_m_ using the same reaction conditions [17]. Nevertheless, the acceptable temperature ranges to discriminate the correct products and artifacts can be, independently, adjusted by the user. The delta peak height (maximum value in negative first derivative to the average value at the inflection points) and width (temperature range between the inflection points) are used to evaluate peak quality. If pre-set minimum cut-offs on peak height and width are not reached, the ‘peak’ is excluded from further analysis. These cut-offs are set to 0.05 of the sum of delta peak heights and 5 °C peak width and can be changed by the user when reproducible low or wide bumps in the negative first derivative should be included or excluded as peaks. Additional files show the melting curve analysis interface [see Additional file 4] and the melting curve analysis results [see Additional file 5]. After melting curve analysis, the program reports the identified peaks per reaction as well as the fraction of the total fluorescence present in the peak of the correct amplification product. The user has two options: either to ignore the result of the melting curve analysis, which is not recommended, or to integrate the results of the melting curve analysis in the amplification curve analysis.

### Integration of melting peak analysis in amplification curve analysis

Apart from identifying reactions that amplify artefacts, the contribution of each melting peak to the total fluorescence in the observed peaks can be used to correct the observed target quantity when artefacts are amplified [17]. To this end, the fluorescence in each observed peak, defined as the fluorescence loss measured in the normalized melting curve between the temperatures of the inflection points of the second derivative, is determined and reported (Fig. 1D). The fractional contribution of the correct peak to the total fluorescence below 1 for the correct peak indicates that, apart from the correct product, one or more artefacts were amplified. When a saturating DNA binding dye is used in the amplification reaction, this fraction can be used as a correction factor to correct the C_q_ and N_0_ found in reactions that show amplification of artifact products [17]. Melting curve analysis thus helps to calculate the true target quantity in these reactions. The application of this correction of the observed C_q_ and N_0_ values is implemented in web-based LinRegPCR and the corrected C_q_ and N_0_ are calculated when the fluorophore allows; the values stored in the RDML file are then updated. The correction factor is stored in the RDML file and the corrected C_q_ and N_0_ values are displayed (Fig. 1E, publication adapted).

### Data import and error reporting via RDML

To enable the analysis of qPCR data, the true raw fluorescence data, not yet baseline-corrected by the qPCR machine software, have to be available in a readable format. The different qPCR machines vendors export these data together with some annotation information in differently formatted text files or spreadsheet tables. To provide a vendor independent, common data format for storage and exchange of qPCR data, the RDML format was introduced into the qPCR field [24, 25]. RDML-Tools support all RDML versions and allow migration between the versions. The LinRegPCR application requires RDML version 1.1 or higher. RDML stores the qPCR information in a zip compressed, predefined XML-formatted text file. After analysis of the qPCR data, RDML supports the storage of PCR efficiency and quantification threshold per assay as well as the C_q_ value and target quantity per reaction. The annotation of the melting temperature is supported from version 1.3 digital PCR onward (http://rdml.org/rdml_v_1_3.html). In RDML, the user has to label each reaction in the run as positive or negative control, or as unknown reaction. This information allows web-based LinRegPCR to provide intelligent error reporting. While processing the amplification data per reaction, the program does a number of quality checks which may lead to warnings and errors reported per reaction (Fig. 2). No amplification in positive controls as well as amplification in negative controls are reported as an error. The warnings, i.e. no amplification, no plateau or deviating PCR efficiency in different sample types, draw the attention of the user to reactions that should be evaluated by eye. The user can then make the choice to exclude these reactions, or the entire assay, from further analyses. This inspection should involve the amplification and melting curves whereas the decision should be based on the purpose of the experiment and the consequences of a false conclusion. E.g. the warning ‘no amplification’ in the positive control is not acceptable in clinical diagnostics, because this occurrence invalidates the conclusion drawn from the absence of amplification in the unknown samples in the run; the whole qPCR run will have to be repeated. However, in experimental research with different tissues among the unknown samples, the concurrent presence of amplification of the correct product in some unknown samples may be enough to accept these results despite the failing positive control. Some errors are too severe to allow the program to automatically calculate the target quantity. However, when the user has reasons to trust the observed C_q_ value, the user can overrule this decision of the program with a manual calculation of the starting concentration using the reported values in ${N}_{0}={N}_{q}/{E}^{{C}_{q},obs}$. The illustration of the program’s flow (Fig. 2) gives guidelines on how to handle the various errors and warnings that are set during processing of the amplification and melting data.

## Results

Our program addresses two types of users: wet-lab scientists wanting to analyze the amplification and melting curves in their own qPCR experiment(s) and bioinformaticians creating pipelines to analyze thousands of such experiments with minimal user involvement. Therefore, the functionality of this web-based application is split into a back-end RDML-Python library for the actual calculations and several companion web applications that visualize the data and provide an interactive access.

### RDML files

To enable machine independent storage and exchange of qPCR data, the RDML format entered the qPCR field in 2009 [24, 25]. This version of LinRegPCR is based on RDML input. Although RDML is available free of charge, not all qPCR machines support the export to RDML files. For analysis of fluorescence data generated by these machines, the user needs to create an RDML file from the text files or spreadsheets exported from their qPCR machine. The RDML-Tableshaper and RDML-Edit tools (see below) help to reformat the various exported files into the common RDML import format that can be used to create an RDML file. An example RDML file can be found in the additional files [see Additional file 6].

### RDML-python library

The stand-alone RDML-Python library (https://github.com/RDML-consortium/rdmlpython) builds the back-end which handles RDML files and performs all amplification and melting curve processing, analyses and calculations. An additional file shows the class design of the Python library [see Additional file 7]. The code is written in Python and depends on the NumPy package (https://numpy.org) for the acceleration of array calculations, the SciPy package (https://www.scipy.org) for advanced statistical calculations and the lxml package (https://lxml.de) for the handling of XML files using the C libraries libxml2 and libxslt. A core functionality of the RDML-Python library can open, read and write RDML files and handle the dependencies within the RDML format. The amplification curve and melting curve analysis parts of the RDML-Python based LinRegPCR are implemented as callable functions linRegPCR() and meltCurveAnalysis() within this library. Results of calculations are written back into the RDML file and, if there are no equivalent elements in the RDML format, displayed as spreadsheet tables. The RDML-Python library can be easily integrated into Python programs and bioinformatics pipelines. It also offers a limited command line interface which can be used to analyze amplification and melting curves in batch programs.

### RDML-tools web applications

The RDML-Tools are hosted on GEAR, a web server for molecular biology applications (https://www.gear-genomics.com/rdml-tools/). The front-end applications are designed for the interactive usage on a molecular biology laboratory floor. The RDML-Tools follow the classic client–server architecture with a web app as front-end calling a dedicated Python server which is performing the calculations using the RDML-Python library. In principle, the web app collects the input files and user input, and sends this information to the server. The server checks the parameters and translates the user requests into calls for the RDML-Python library. Once the calculations are complete, the server sends the results back to the web app which displays the results and allows the user to explore them.

The www.gear-genomics.com server stores the data for a maximum of 3 days after analysis. Only the user can excess the data during this period by a unique id. Users may, however, choose to permanently delete their uploaded data immediately after analysis following the "Remove Uploaded Data from Server" link on the main tab of the program. The RDML-Tools are available under GPL license as source distribution for the installation on private servers (https://github.com/RDML-consortium/rdml-tools) and are explained below.

### RDML-tools help

RDML-Tools Help is available on the RDML-Tools page and provides an introduction to the RDML-Tools and gives detailed information on the parameters of the tools and their optimal usage.

### RDML-validate

XML files are bound to a predefined usage of elements by schema files. RDML-Validate validates RDML files against the RDML schema of the correct version. The tool will show a report including the used RDML version. If errors are encountered, a hint to the conflicting rule is given. RDML-Edit (see below) can then be used to correct the error.

### RDML-TableShaper

Not all qPCR machines export RDML files and, therefore, many users are left with plain spreadsheet exports that cannot be directly imported into RDML-Edit (see below). RDML-TableShaper (https://www.gear-genomics.com/rdml-tools/tableshaper.html) fills the gap by loading the exported spreadsheets and converting them step by step into the format that can be imported into RDML-Edit for further editing and annotation. If the export format of the qPCR machine is already known, a file with the correct parameters can be selected from a dropdown menu. Otherwise, these parameters must be found by iteratively walking through the tabs of RDML-TableShaper. Once completed, the import parameters can be saved for future use. An additional file shows an example of the TableShaper interface [see Additional file 8].

### RDML-edit

RDML-Files can store a multitude of data. RDML-Edit allows to view and edit RDML files by focusing on a user selected part of the file, which is displayed in the active tab. To avoid accidental modification, RDML-Edit by default only displays the data. If the edit-mode is activated, all elements can be modified and additional information can be included, except for the RDML elements containing the raw fluorescence data of the qPCR as there is no valid reason the change these data. In order to use the LinRegPCR application, the user needs to use RDML-Edit and RDML-Tableshaper to annotate the targets, dye, samples, sample type and reactions, when this information is not yet present in the qPCR machine output. For proper functioning, it is essential that the sample type, positive and negative controls, as opposed to the unknown samples are annotated. Note that RDML-Edit can also convert between different RDML versions.

### RDML-RunView

RDML-Edit shows all information up to the single run. Once a single run is selected in RDML-Edit, it is visualized in RDML-RunView in two ways. One view shows the plate lay-out with information about the sample, target and C_q_ (if called and exported by the qPCR machine), the other view shows the fluorescence data as a graph. The user can choose between amplification and melting curves, customize the used colors, for example to discriminate between wells with different targets or different tissue samples and switch between logarithmic and linear scaling of the fluorescence axis. An additional file shows an example of the RunView interface [see Additional file 9].

### RDML-LinRegPCR

RDML-LinRegPCR facilitates visualization and analysis of amplification and melting curve data of a single qPCR run. The user interface is similar to RDML-RunView and is used to display the contents of the RDML file or the analysis results on the Amplification Curve Analysis and Melting Curve Analysis tabs. The Amplification Curve Analysis tab allows to (re)calculate C_q_ values using the LinRegPCR algorithm. The results are displayed in a spreadsheet table and deviant reactions are highlighted. If a row is double-clicked, the amplification curve of the corresponding reaction is highlighted in the RunView tab complemented with the window of linearity, and the values of the quantification threshold and C_q_. The Melting Curve Analysis tab shows the results from the melting curve analysis. Screenshots of the amplification curve analysis and melting curve analysis interfaces are shown in additional files [see Additional file 2 to 5]. The results in the spreadsheet tables can be saved as CSV files or exported to programs like Microsoft Excel or Libre Office Calc for calculation of gene-expression ratios, fold-change between experiments and statistical analysis. Graphs can be exported as SVG, pasted into presentation programs, like PowerPoint, or modified in vector programs, like Impress and Inkscape. Figure 1A,B were created from such exported SVG files using Inkscape. Figure 1C–E are edited examples of the table views on the analysis tabs exported as CSV files.

## Discussion

Most qPCR machine software does not extract all relevant information that is present in the amplification curves. Because reaching the quantification threshold is often enough to consider a reaction to be positive, this also results in the assignment of a C_q_ value to a low-quality reaction. Although the outcome of such reactions could be of relevance in clinical diagnostics, they should not be used for quantitative purposes, because low quality is often associated with a very low PCR efficiency [3]. The amplification curve analysis implemented in LinRegPCR provides qualitative and quantitative details on each individual reaction and thus enables the detection of deviating reactions, assays and runs. Because the baseline estimation of LinRegPCR is based on the data points in the exponential phase and does not use the early cycles of the PCR, it is not affected by random ground phase noise. This unique baseline estimation algorithm might explain why LinRegPCR achieved qPCR results with the lowest variation and highest reproducibility in a comparison of amplification curves analysis approaches [14].

For calling the C_q_ value of individual reactions, the LinRegPCR web-application uses the mean PCR efficiency per assay. In doing so, the reported C_q_ value is not affected by residual random baseline noise. Although LinRegPCR thus reports C_q_ values called from a common quantification threshold per run, which would allow direct comparison of C_q_ values, it is not recommended to do so. Because of the dependency of C_q_ on the PCR efficiency, conclusions drawn from ΔC_q_ values can be severely biased [2, 3, 5]. Only efficiency-corrected qPCR results can be compared and reproduced between studies.

By default, LinRegPCR calculates the target quantity per reaction using the PCR efficiency per assay. This efficiency per assay is calculated as the mean of the PCR efficiencies observed for the individual reactions per assay. The reporting of the PCR efficiency for individual reactions in the output table not only serves as a quality criterion but this PCR efficiency value can also be used to calculate the starting concentration of the individual reaction. The latter option is often indicated in case of clinical point-of-care analysis, where sample purification can be less optimal, or even absent, and the PCR efficiency can be affected by sample contaminations. In those cases, the variation in the PCR efficiencies between samples does not allow calculation of a reliable and meaningful PCR efficiency per assay [3]. Because LinRegPCR reports the C_q_ value and PCR efficiency of the individual reaction in the output, the user can manually calculate the starting concentration of individual reactions using the reported values.

Although many researchers are not aware of the existence of the melting curve analysis, this analysis should be considered an essential step in qPCR analysis, especially when DNA-binding dyes are used to monitor the PCR. The melting curve analysis allows the identification of artefacts besides the intended target, which is easy, quick, cheap and more sensitive than size separation on agarose gels [16, 17]. The example in Fig. 1A,B shows the same reaction with an input of an artificial mix with 75% artifact and 25% correct amplicon. The amplification curve is perfect, passes all quality controls and would not be rejected (Fig. 1A). Only the melting curve analysis reveals the presence of artifacts (Fig. 1B) and provides the user with an error warning. This example shows that, when the researcher does not identify and exclude reactions in which an artefact is synthesized, the reported quantification result is meaningless.

This new version of LinRegPCR includes a user-independent analysis of the melting curve data and reports all observed melting peaks. The program identifies the peak of the intended amplification target using the melting temperature given in the RDML input. To this end, the user has to determine this melting temperature from a pilot study with positive control samples. In a validated PCR assay, such a positive control should show amplification of only one product. The melting curve analysis primarily serves to determine whether the correct product, artefacts or both are amplified in unknown samples. Customarily, reactions that amplify artefacts are excluded from further analysis. However, we recently showed that the results of the amplification curve analysis can be corrected by determining the contribution of the correct peak to the total fluorescence [17]. This means that, based on the melting curve analysis, in a reaction that also amplifies (an) artefact(s), the intended target can be correctly quantified. Implementing this correction means that these reactions do no longer have to be discarded and are not lost from the study. The latter correction is only possible when a saturating DNA-binding dye, like LCGreen, is used [17].

When to use the LinRegPCR program? The amplification curve analysis in LinRegPCR can be used for analysis of qPCR data resulting from all amplification monitoring modalities [26]. Melting curve analysis requires DNA-binding dyes or hybridisation probes, that bind to the double strands DNA and are released during heating [16]. Correction of the observed C_q_ or N_0_ with the melting curve results requires that the observed fluorescence comes from a saturating dye [17].

How to use results of this program? The main result of the analysis of qPCR data with LinRegPCR is the target quantity (N_0_) per reaction, calculated with the quantification threshold per run, PCR efficiency per assay and C_q_ value per reaction. These target quantities represent the gene expression per reaction, and can be used to calculate the gene-expression ratio between target and reference genes (ratio = N_0,target_/N_0,reference gene_) and the fold-difference between experimental conditions (fold = ratio_experiment_/ratio_control_). After analysis of the amplification curves the annotated results (efficiency per assay and C_q_ per reaction) are stored in the RDML file. These analysis results can be further analysed with specialised programs like qBase [27] or exported to Excel for additional calculations, presentations and statistical analysis.

As described, all tools involved in this Python-based version of LinRegPCR are open source and the tools are free to use. The implementation of the algorithms is transparent and can be extended by the users’ requirements. The RDML-Python library is available under MIT license as source distribution (https://github.com/RDML-consortium/rdmlpython) or as 'rdmlpython' package using pip3. During implementation of the different functions of this web-based LinRegPCR some changes were also made in the Windows version LinRegPCR. These changes did not affect the target quantities reported by the Windows version. Although the different quantification thresholds result in different C_q_ values, upon release of the web-based LinRegPCR, the reported target quantities (N_0_) of both program versions are identical [see Additional file 1]. Although the reported C_q_ values are based on a common threshold, we do not recommend direct comparison of these C_q_ values without taking the PCR efficiency into account [3] [21]. The web-based version of LinRegPCR is platform-independent and its processing time is roughly six times faster than the Windows version. The warning and error report of the web-based version, partly based on the information in the RDML input file, is more extensive and reactions with an aberrant PCR efficiency are identified with a statistical evaluation without user input. The integration of melting curve analysis allows automatic identification and exclusion of reactions in which artifacts are amplified. After release of web-based LinRegPCR, the Windows version will stay available and its use will be supported. However, it will no longer be updated.

## Conclusions

The web-based version of LinRegPCR was developed to overcome the limitations of the original programs for amplification curve analysis [6, 13] and melting curve analysis [17]. Apart from the significant increase in processing speed, the web implementation of these analysis programs provides platform independence. With the inclusion of a statistical outlier detection option and the integration of melting curve analysis, this new version of LinRegPCR is a comprehensive analysis tool for analysis of qPCR data resulting in quantitative results as well as quality checks based on amplification and melting curve data. The analysis results are stored in the RDML-file and summarized in a spreadsheet format that can be exported for further analysis. Similarly, the graphs can be exported in a vector graphics format that allows easy formatting in other programs.

### Availability and requirements

- **Project name**: RDML-Tools
- **Operating system(s)**: Platform independent
- **Programming language**: Python, Java Script
- **Other requirements**: numpy, scipy, lxml, flask
- **License**: GPL-3.0 (RDML-Tools), MIT (RDML-Python library)
- **Any restrictions to use by non-academics**: none

## Supplementary Information

**Additional file 1.** Excel file comparing the output of the new Python with the original Windows LinRegPCR version. Also illustrating the escence axis.

**Additional file 2.** Screenshot of the LinRegPCR web interface showing the amplification curve analysis RunView tab.

**Additional file 3.** Screenshot of the LinRegPCR web interface showing the LinRegPCR tab with results of the amplificationcurve analysis.

**Additional file 4.** Screenshot of the MeltCurveAnalysis web interface showing the melting curve analysis RunViewtab.

**Additional file 5.** Screenshot of the MeltCurveAnalysis web interface showing the MeltCurveAnalysis tab withresults of the melting curve analysis.

**Additional file 6.** This file contains example amplification and melting curve data in RDML format.

**Additional file 7.** Overview of the classes defined in the RDML-Python library..

**Additional file 8.** Screenshot of the TableShaper web interface showing an example conversion form spreadsheettot RDML format.

**Additional file 9.** Screenshot of the RunView web interface showing the plate-layout with reaction annotation andraw fluorescence data on a logarithmic fluorescence scale.

## Data Availability

All data generated or analysed during this study are included in this published article and its supplementary information files. The source code of the applications presented in the current study are available in the RDML-consortium repositories, https://github.com/RDML-consortium.

## Abbreviations

qPCR: Quantitative PCR
PCR: Polymerase chain reaction
C_q_: Quantification cycle
ΔC_q_: Difference between two C_q_ values
N_q_: Quantification threshold
N_0_: Starting concentration or target quantity
E: PCR efficiency
W–o-L: Window of linearity
T_m_: Melting temperature
RDML: Real-time PCR data markup language
SDM: Second derivative maximum
CV: Coefficient of variation
