# Supplementary figures and images for: Web-based LinRegPCR: application for the visualization and analysis of (RT)-qPCR amplification and melting data
Source: BMC Bioinformatics. 2021 Aug 24;22:398. doi: 10.1186/s12859-021-04306-1 (PMC8386043; doi:10.1186/s12859-021-04306-1)

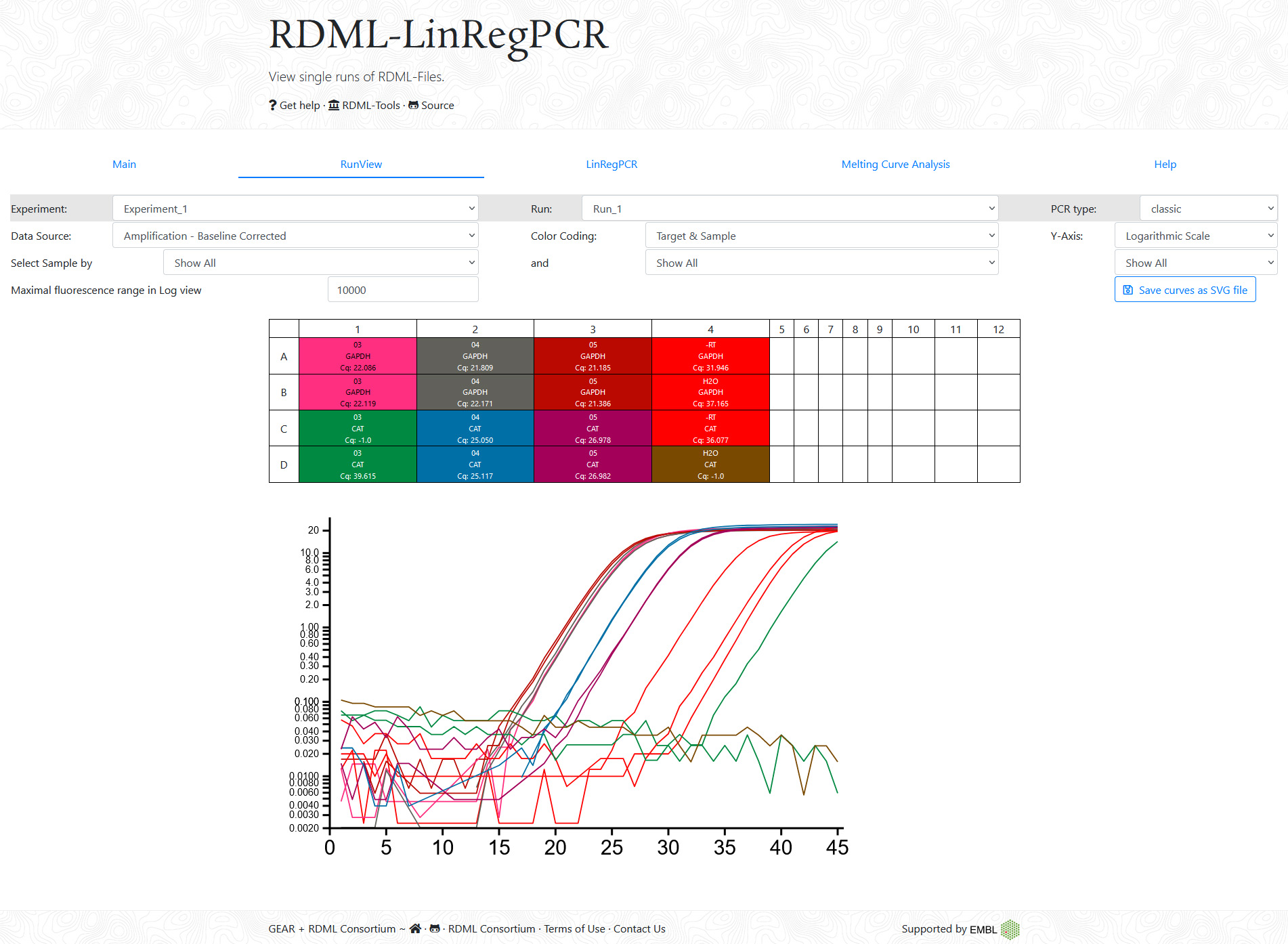

Supplement: Supplementary file 2 — Additional file 2. Screenshot of the LinRegPCR web interface showing the amplification curve analysis RunView tab. [file 12859_2021_4306_MOESM2_ESM.jpg]

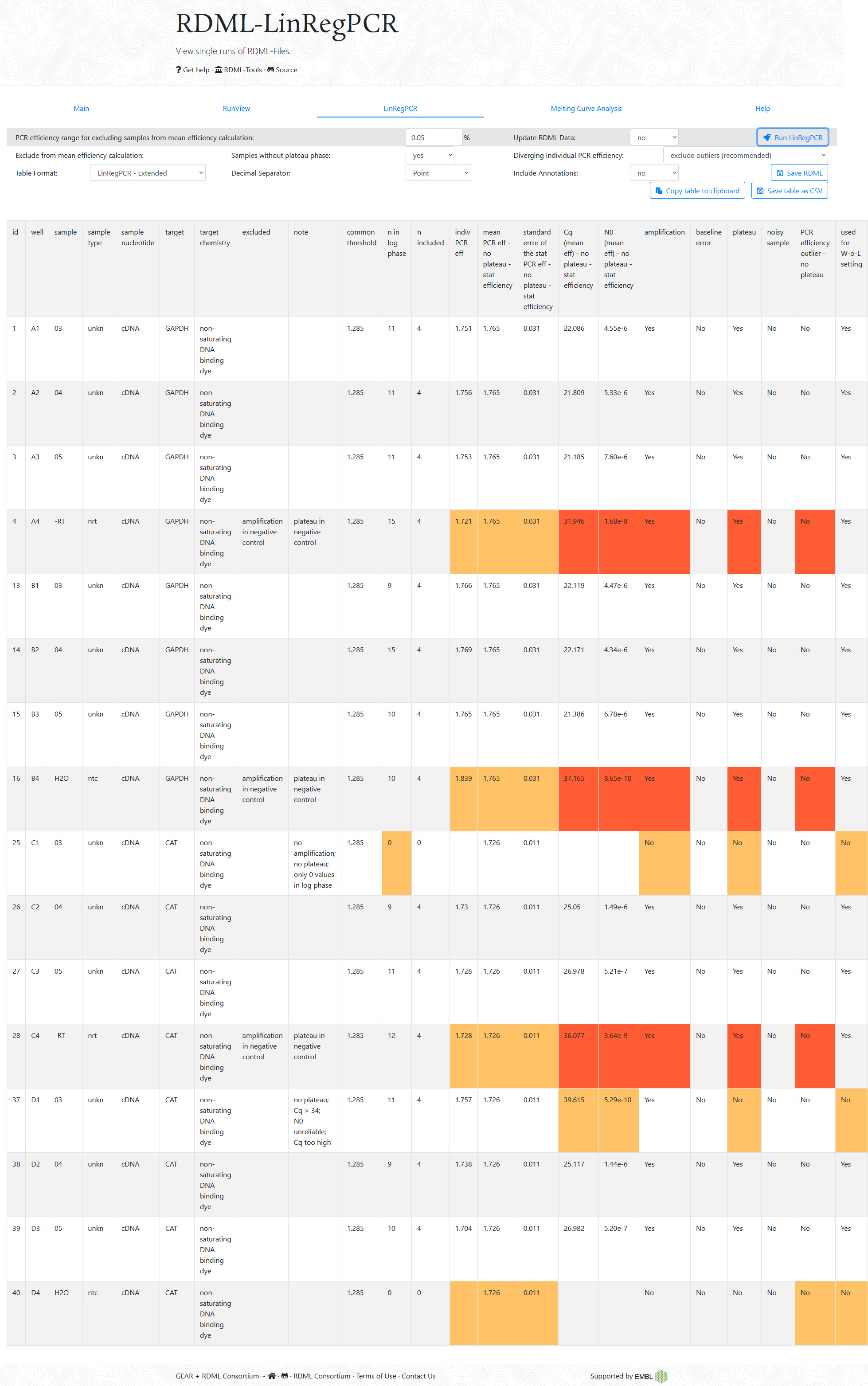

Supplement: Supplementary file 3 — Additional file 3. Screenshot of the LinRegPCR web interface showing the LinRegPCR tab with results of the amplificationcurve analysis. [file 12859_2021_4306_MOESM3_ESM.jpg]

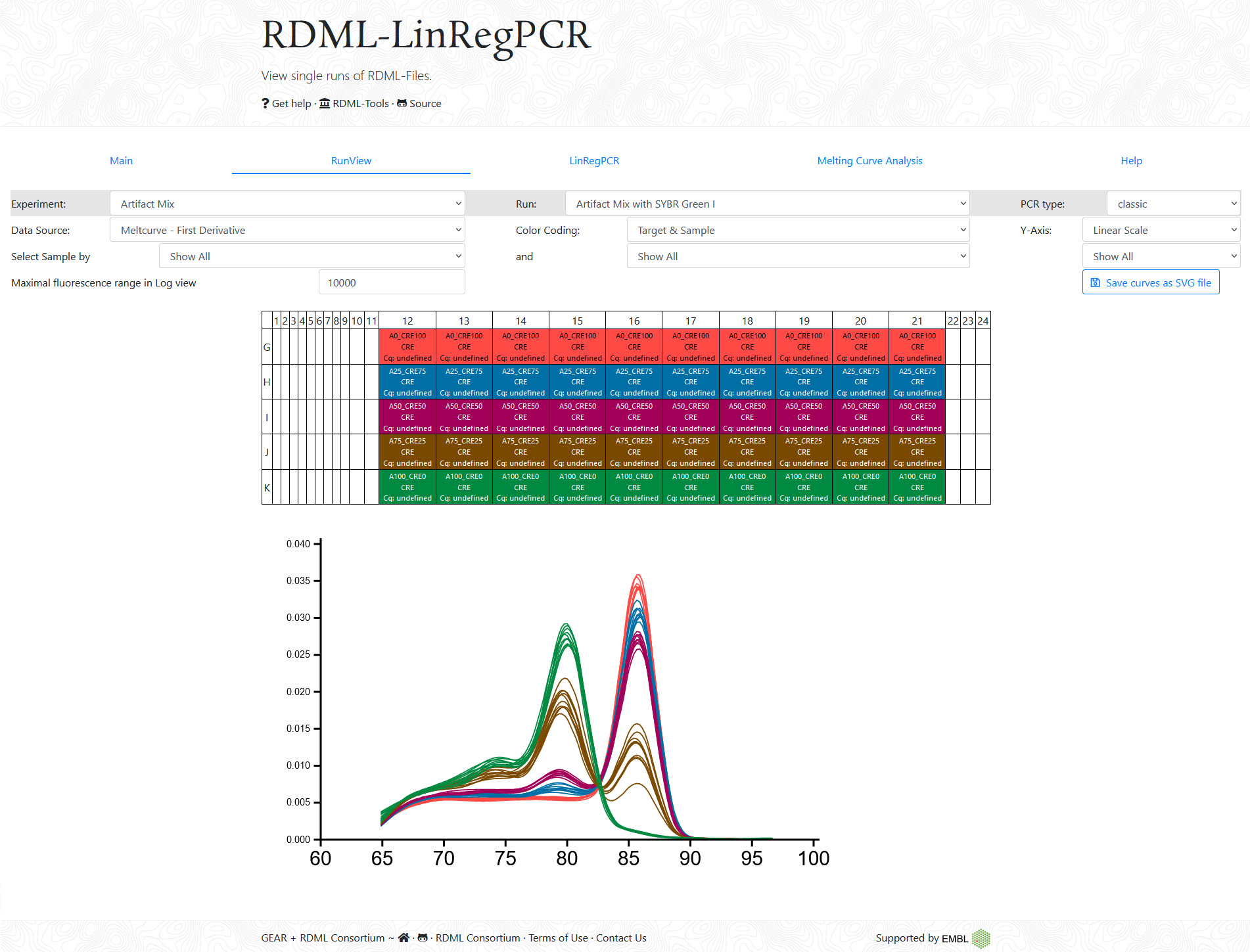

Supplement: Supplementary file 4 — Additional file 4. Screenshot of the MeltCurveAnalysis web interface showing the melting curve analysis RunViewtab. [file 12859_2021_4306_MOESM4_ESM.jpg]

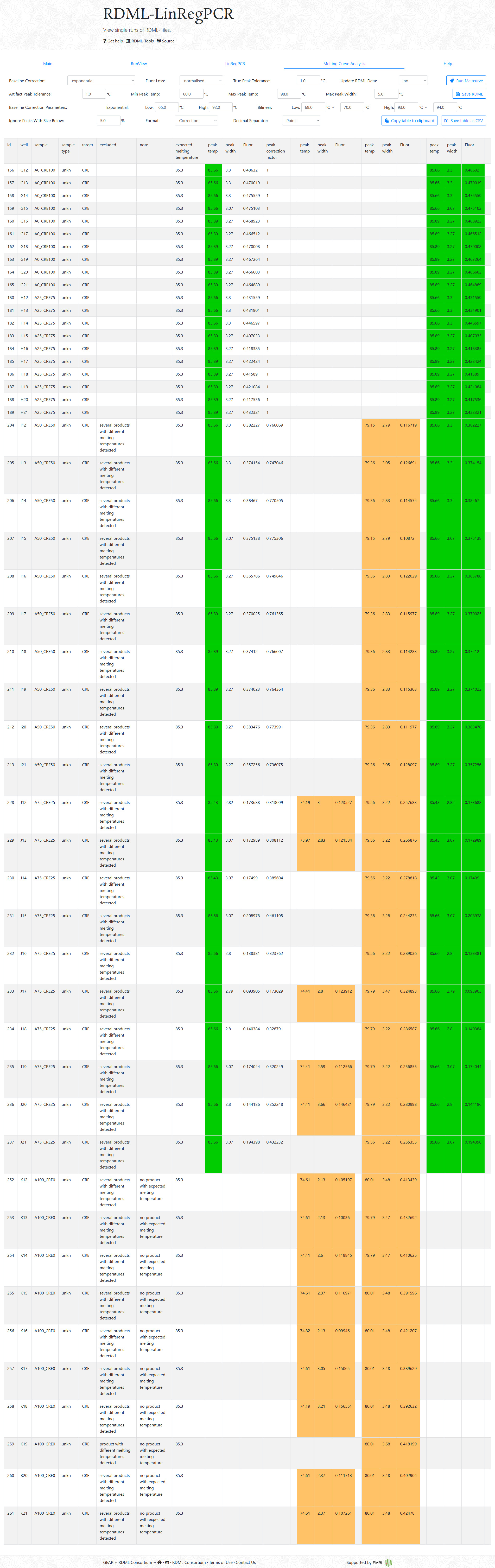

Supplement: Supplementary file 5 — Additional file 5. Screenshot of the MeltCurveAnalysis web interface showing the MeltCurveAnalysis tab withresults of the melting curve analysis. [file 12859_2021_4306_MOESM5_ESM.jpg]

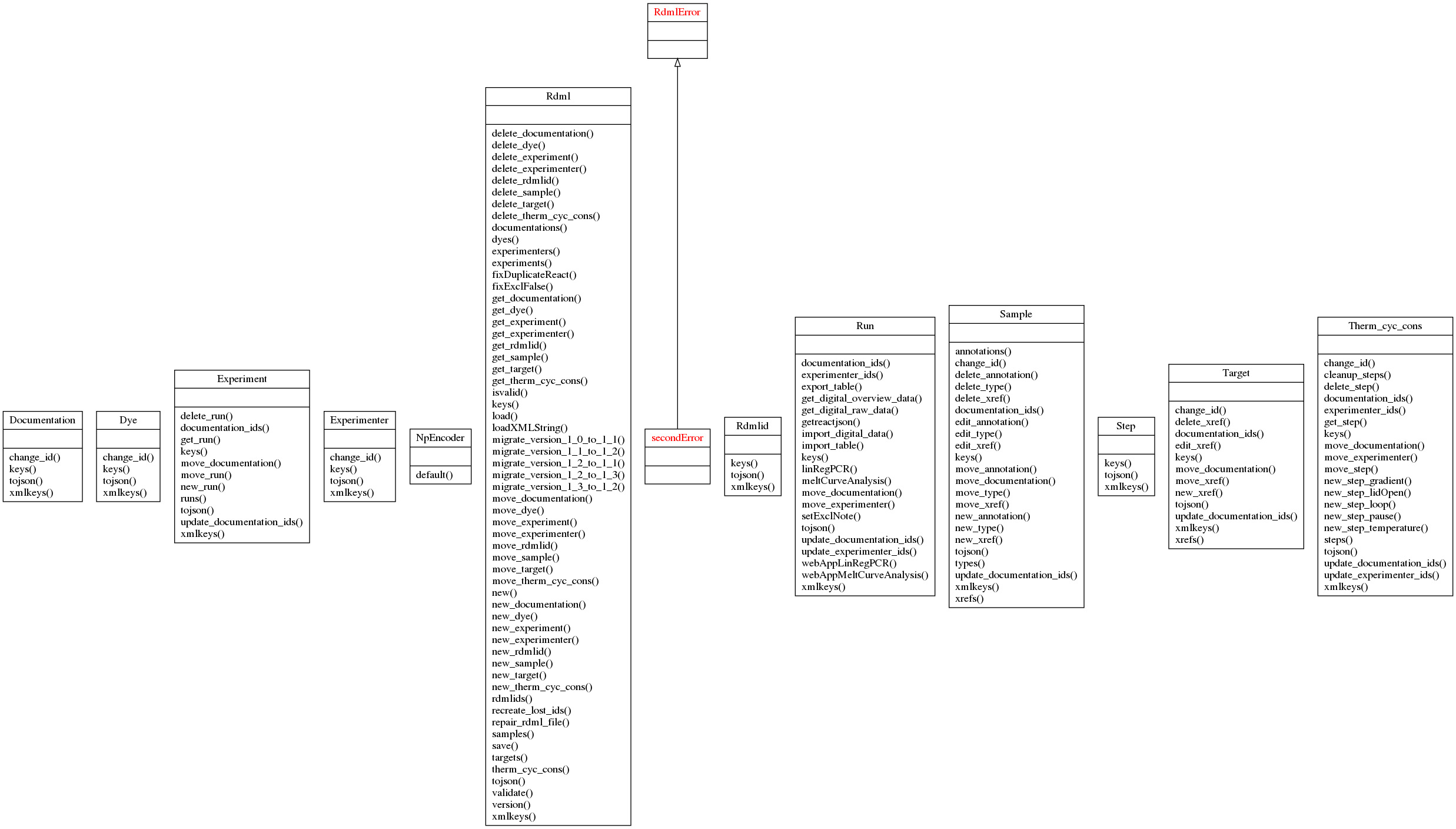

Supplement: Supplementary file 7 — Additional file 7. Overview of the classes defined in the RDML-Python library.. [file 12859_2021_4306_MOESM7_ESM.jpg]

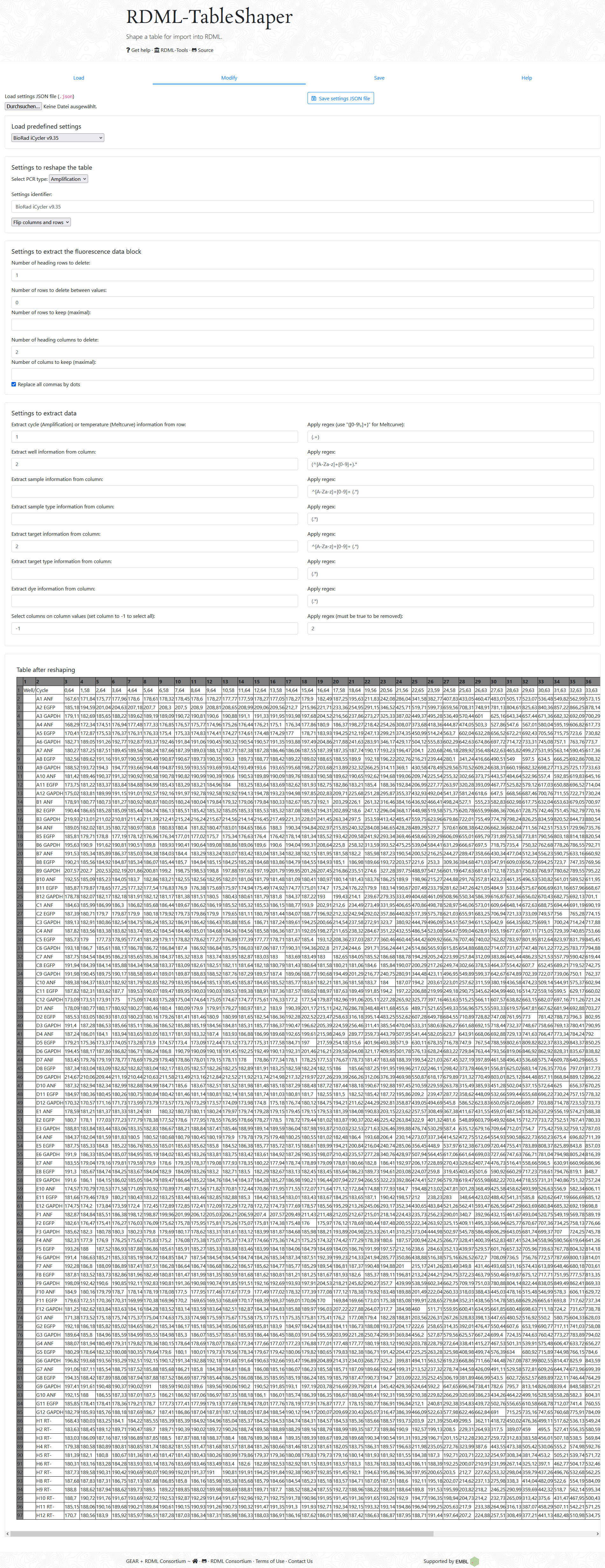

Supplement: Supplementary file 8 — Additional file 8. Screenshot of the TableShaper web interface showing an example conversion form spreadsheettot RDML format. [file 12859_2021_4306_MOESM8_ESM.jpg]

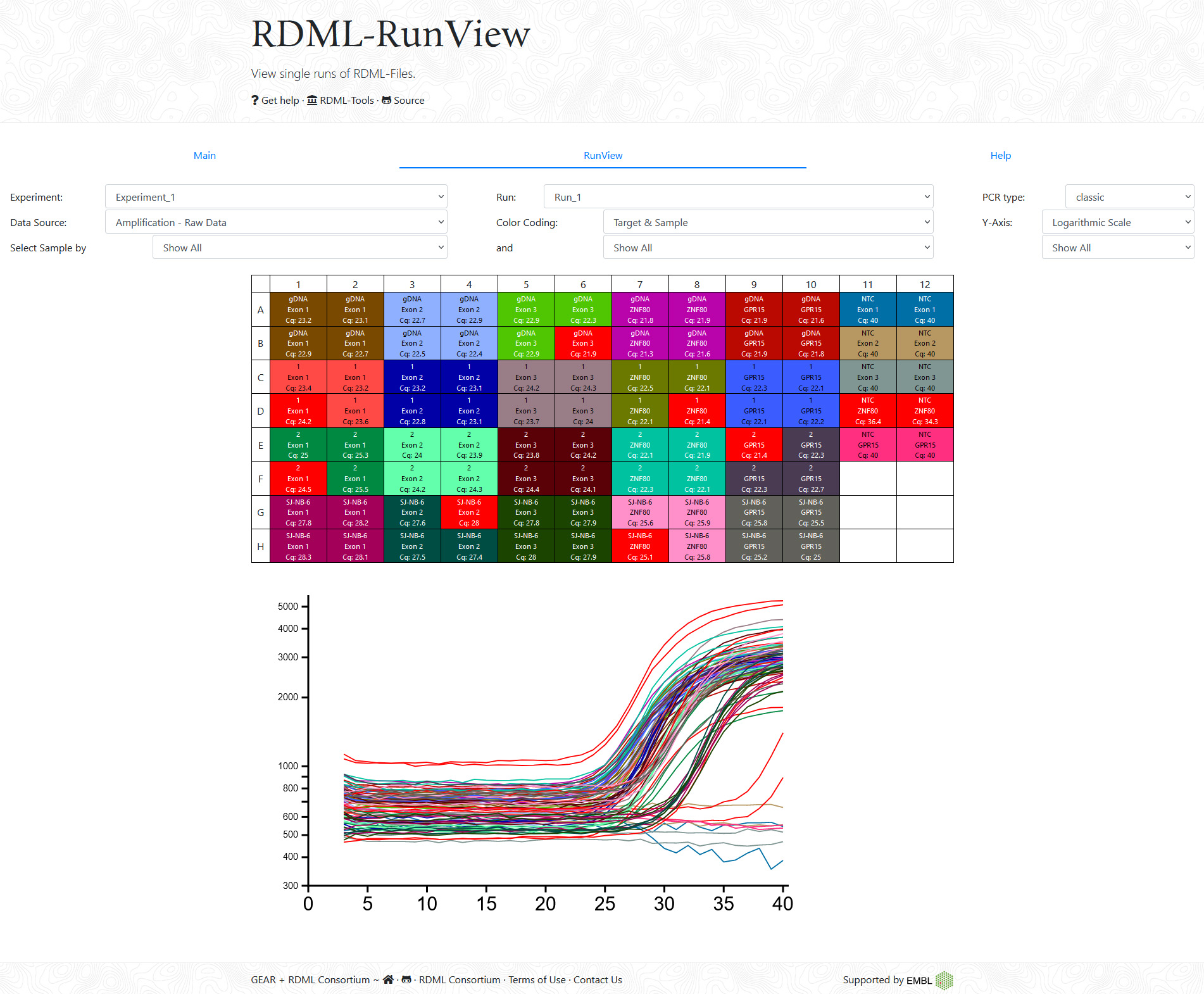

Supplement: Supplementary file 9 — Additional file 9. Screenshot of the RunView web interface showing the plate-layout with reaction annotation andraw fluorescence data on a logarithmic fluorescence scale. [file 12859_2021_4306_MOESM9_ESM.jpg]
